# Receptor-Binding and Oncogenic Properties of Polyoma Viruses Isolated from Feral Mice

**DOI:** 10.1371/journal.ppat.0030179

**Published:** 2007-12-14

**Authors:** John Carroll, Dilip Dey, Lori Kreisman, Palanivel Velupillai, Jean Dahl, Samuel Telford, Roderick Bronson, Thomas Benjamin

**Affiliations:** 1 Department of Pathology, Harvard Medical School, Boston, Massachusetts, United States of America; 2 Department of Tropical Public Health, Harvard School of Public Health, Boston, Massachusetts, United States of America; University of Wisconsin-Madison, United States of America

## Abstract

Laboratory strains of the mouse polyoma virus differ markedly in their abilities to replicate and induce tumors in newborn mice. Major determinants of pathogenicity lie in the sialic binding pocket of the major capsid protein Vp1 and dictate receptor-binding properties of the virus. Substitutions at two sites in Vp1 define three prototype strains, which vary greatly in pathogenicity. These strains replicate in a limited fashion and induce few or no tumors, cause a disseminated infection leading to the development of multiple solid tumors, or replicate and spread acutely causing early death. This investigation was undertaken to determine the Vp1 type(s) of new virus isolates from naturally infected mice. Compared with laboratory strains, truly wild-type viruses are constrained with respect to their selectivity and avidity of binding to cell receptors. Fifteen of 15 new isolates carried the Vp1 type identical to that of highly tumorigenic laboratory strains. Upon injection into newborn laboratory mice, the new isolates induced a broad spectrum of tumors, including ones of epithelial as well as mesenchymal origin. Though invariant in their Vp1 coding sequences, these isolates showed considerable variation in their regulatory sequences. The common Vp1 type has two essential features: 1) failure to recognize “pseudoreceptors” with branched chain sialic acids binding to which would attenuate virus spread, and 2) maintenance of a hydrophobic contact with true receptors bearing a single sialic acid, which retards virus spread and avoids acute and potentially lethal infection of the host. Conservation of these receptor-binding properties under natural selection preserves the oncogenic potential of the virus. These findings emphasize the importance of immune protection of neonates under conditions of natural transmission.

## Author Summary

Strains of the mouse polyoma virus adapted to growth in cell culture vary greatly in their abilities to cause disease. Pathogenicities of these laboratory strains range from “attenuated” to “highly virulent” when tested in animals. The biological differences are based in large part on variations in the outer capsid protein, which dictate the manner in which the virus recognizes and binds to cell receptors. In contrast, strains of virus newly isolated from wild mice are uniform in their receptor-binding properties. Naturally occurring strains avoid binding to pseudoreceptors, which would severely limit their ability to spread. At the same time, their avidity of binding to true receptors is sufficiently strong to avoid rapid dissociation and potentially lethal spread. They are therefore neither attenuated nor virulent. The new isolates do, however, retain the ability to induce a broad spectrum of tumors in the laboratory. These findings emphasize the importance of neonatal and maternal immune responses in allowing a potentially highly oncogenic virus to disseminate without causing disease.

## Introduction

Interest in the murine polyoma virus (Py) grew out of its discovery as a tumor-inducing agent in its natural host under experimental conditions [1]. The original virus isolates were obtained from tissues of inbred laboratory mice [2]. Plaque purification and molecular cloning have been used in different laboratories to establish independent “wild-type” virus strains, which have been propagated in cell culture over many years. Studies of these strains have provided a detailed understanding of the replicative and transforming functions of the virus [3], as well as their tumorigenic properties [4]. Laboratory strains have also been highly purified and used for structural studies of the virus and its interaction with receptors [5].

Cell transformation based on focus formation or growth in soft agar has generally been accepted as the in vitro counterpart of tumor induction in the animal. However, assays in cell culture do not give a complete account of functions required by the virus to replicate or induce tumors in mice. Py mutants altered in tumor (T) antigen functions essential for transformation may retain the ability to induce tumors [6,7]. Moreover, different “wild-type” virus strains commonly used in the laboratory, while equally efficient in transforming cells in vitro*,* may differ greatly in their abilities to replicate and induce tumors in the intact host [8]. These differences are related not to T antigen functions but rather to receptor-binding properties of the major capsid protein Vp1.

Structural, genetic, and biological studies have come together to provide an understanding of how Vp1 polymorphisms affect virus binding to sialic acid moieties as an essential component of cell receptors [4,5,9,10]. Single amino acid substitutions at two positions in Vp1 alter the selectivity and avidity of virus binding to sialic acid [5,11,12]. These polymorphisms have little or no effect on the behavior of the virus in cell culture [13,14] but have profound effects in the animal [10,14,15].

Despite its pathogenic potential in the laboratory, Py establishes a silent infection in nature. Virus shed from persistently infected mothers is transmitted to neonates which maintain the virus for life without developing tumors [2,16,17]. In contrast, when inoculated into newborn mice from a virus-free colony, as little as a few plaque-forming units of an appropriate virus strain is sufficient to induce the development of multiple tumors ([8] and unpublished data). The absence of pathogenic effects in naturally infected mice suggests that virus strains found in nature might correspond to non– (or low) tumor-inducing strains. It is also possible that more aggressively replicating strains with potential to induce tumors or to kill the host outright may be found in nature, their pathogenic effects offset by protective antibody delivered through the milk and by rapidly maturing immune responses of the pups. Differences in host genetic backgrounds between inbred laboratory mice and feral mice may also contribute to the different outcomes.

Because laboratory strains of virus have been propagated and manipulated in culture over many years, it is unclear how their properties relate to those of naturally occurring virus strains. This investigation was undertaken to determine the Vp1 types and pathogenic properties of truly wild-type strains of Py. To this end, we trapped feral mice at multiple locations and screened them serologically for Py. Independent isolates of the virus were established from seropositive animals. Vp1 coding sequences and biological properties of these isolates have been compared with prototype laboratory strains.

## Results

### Major Determinants of Pathogenicity Lie in the Sialic Acid Binding Pocket of Vp1

**Figure 1:**
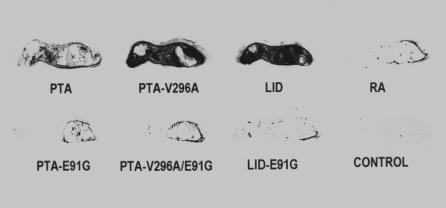
Effects of Single Amino Acid Replacements in Vp1 on Replication and Spread in Neonatally Infected C3H/BiDa Mice Whole mouse section hybridizations were carried out one week after virus inoculation (dose of 2 − 5 × 10^7^ pfu/animal). See Table 1 and text. Reprinted from [10] with permission from Academic Press.

Properties of three prototype “wild-type” laboratory strains of Py are summarized in Table 1. The abilities of these strains and of several mutants derived from them to replicate in newborn mice were previously compared by whole mouse section hybridization (Figure 1). The small plaque strain RA shows limited replication and induces few or no tumors. Tumors that do arise are strictly of mesenchymal origin and develop after a long latency approaching one year [8]. The large plaque strain PTA gives rise to a disseminated infection followed by development of multiple tumors that are of epithelial as well as mesenchymal origin. Tumors may be detected grossly as early as 5–6 wk and grow rapidly, leading to a moribund condition usually within 2–3 mo in essentially 100% of the animals [8]. LID, a large plaque strain derived from PTA, is virulent [18], killing neonates within a few weeks due to rapid dissemination and destruction of vital tissues [9,19]. The LID strain has acquired virulence without losing tumorigenicity, because mice inoculated with low doses may survive and develop tumors [9].

**Table 1:**
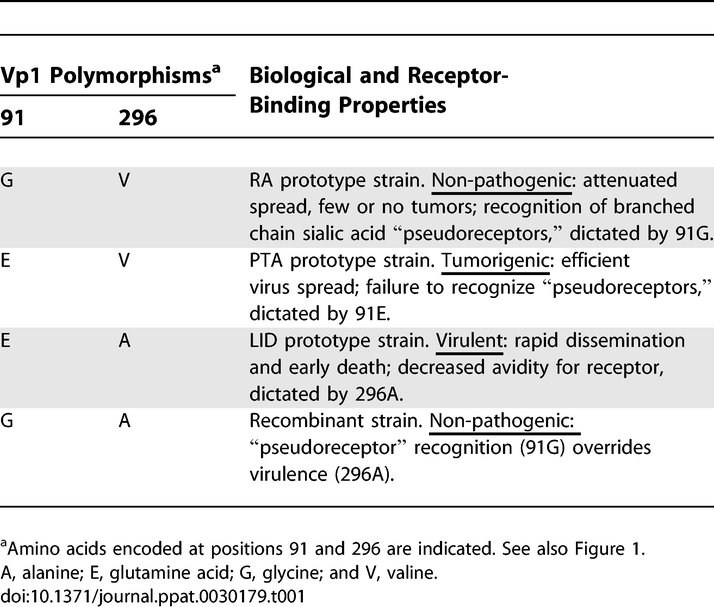
Determinants of Pathogenicity in the Sialic Acid Binding Pocket of Vp1

The different levels of pathogenicity of these three strains are governed to a large extent by their Vp1 type [9,14]. Amino acid substitutions at positions 91 and 296 are critical in determining receptor recognition properties of the virus [5,9]. The common receptor(s) for all Py strains contain a terminal sialic acid (NeuNAc) in an α-2,3 linkage to galactose. Certain gangliosides carrying this linkage have been shown to be functional receptors, binding and internalizing the virus and conveying it to the endoplasmic reticulum for disassembly and translocation [20–23]. The sialic acid binds in a shallow groove of complementary shape on the surface of the virus with no apparent conformational changes induced in Vp1 upon binding. Co-crystal structures of whole virus or recombinant Vp1 pentamers together with sialyloligosaccharides have been determined at high resolution. These reveal electrostatic, hydrogen bond, and van der Waals interactions between side chains of Vp1 and the oligosaccharide receptor. Substitutions of glycine for glutamic acid at position 91 and alanine for valine at position 296 have profound effects, the former in allowing the virus to discriminate between true receptors and “pseudoreceptors” and the latter in determining binding affinity and ease of dissociation [5,11,12].

The effects of these substitutions can be rationalized as follows. The carbohydrate moiety of the receptor is commonly found in a trisaccharide [NeuNAc-(α2,3)-Gal-(β1,3)-GalNAc] in which the GalNAc in the third position may be linked via an α2,6 linkage to another sialic acid, forming a branched di-sialic acid chain [NeuNAc-(α2,3)-Gal-(β1,3)-(NeuNAc-(α2,6)-GalNAc]. Small plaque strains such as RA bind to both the branched and straight chain oligosaccharides, while large plaque strains bind only to the straight chain [24,25]. The crystal structures indicate that electrostatic repulsion and steric interference between the glutamic acid at position 91 of Vp1 and an α2,6-linked sialic acid would prevent virus binding to the branched chain oligosaccharide. For this reason, large plaque strains such as PTA and LID with glutamic acid at 91 are constrained to bind only the straight chain receptor. Substitution of glycine for glutamic acid at this position opens up another pocket able to accommodate the second (α2,6-linked) NeuNAc. RA and other small plaque strains are thus able to bind both the branched and the straight chain oligosaccharides. Because small plaque strains have broader receptor binding capability and at the same time are inhibited in replication in the mouse (compare RA and PTA in Figure 1), the branched chain oligosaccharide is considered to be a pseudoreceptor, i.e., one that binds virus without leading to infection and thus retarding virus spread [5,10]. The polymorphism at position 91 also dictates plaque size, presumably by affecting release from cell debri and relative ease of spread in cell monolayers. It also affects interaction with erythrocyte receptors, altering the pH- and temperature-dependence of hemagglutination [13].

The valine to alanine substitution at position 296 introduces a subtle alteration in a hydrophobic surface forming part of the sialic acid binding groove. Valine at this position (PTA) makes a van der Waals contact with the sialic acid ring, while the shorter chain alanine (LID) does not. The alanine substitution thus weakens the hydrophobic interaction with the receptor [9]. Loss of this interaction is expected to facilitate dissociation of the virus from cell debri following a lytic infection. The ability of LID to spread rapidly and destroy vital tissues underlies its virulent behavior. Site-directed mutagenesis has confirmed the importance of these substitutions (Figure 1 and [10]). Thus, PTA-V296A spreads more rapidly and extensively than PTA and to a degree similar to LID. It also induces early death in neonatal mice [10]. Studies of site-directed mutants have also shown that recognition of branched chain pseudoreceptors exerts a dominant effect biologically. Introduction of 91G into virulent or tumorigenic strains results in attenuation of spread. This is seen by comparing PTA-E91G, PTAV296A-E91G, and LID-E91G with their respective parental strains (Figure 1). PTA-E91G has been shown to be less tumorigenic than PTA, consistent with its reduced ability to spread [10].

### Screening of Feral Mice and Isolation of Py

To determine the Vp1 type(s) of Py under natural conditions, feral mice were trapped and screened serologically for ones that carried the virus. A total of 71 mice were trapped alive from 12 locations in and around Boston and as far away as Cuttyhunk Island, MA, and New York City. Mice were humanely killed, sera collected, and kidneys frozen for attempts at virus isolation. Screening for Py was carried out on the serum from each mouse using a hemagglutination inhibition (HA-I) assay. Results are shown in Table 2. Using a cutoff of 160 HA-I for non-specific inhibition, mice fell into two groups based on their HA-I titers and the location where they were trapped. Mice from four locations were uniformly negative, with HA-I titers ranging from 20 to 160. Mice trapped at the other eight locations were all positive, with HA-I titers ranging from 640 to 10,240. Eight locations yielded multiple mice. Mice from a given location were either all positive or all negative indicating that whenever present the virus spreads efficiently within each breeding population. No tumors were noted in either the HA-I–positive or –negative mice, with one exception, a fibroma, which upon histological examination appeared not to be typical of Py tumors. These findings of efficient spread with no discernible pathology are consistent with earlier reports on the epidemiology of natural infections by Py [16,26,27].

**Table 2:**
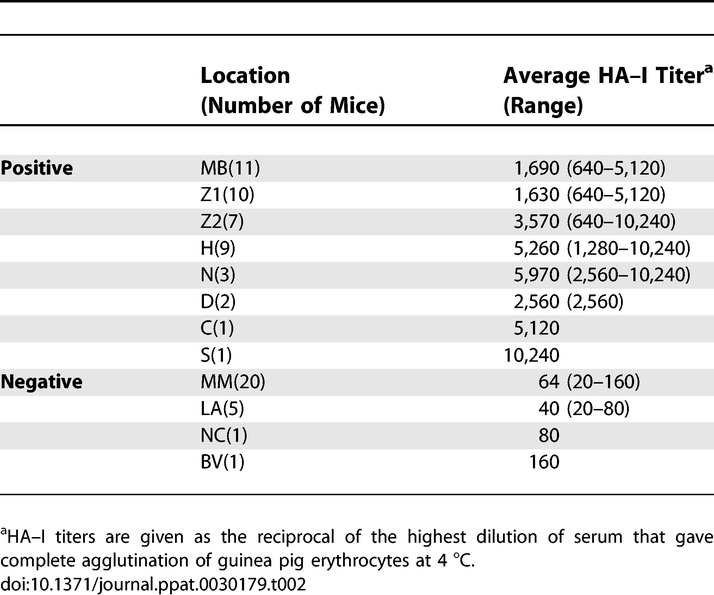
Serological Tests for Py Virus in Feral Mice

Py was isolated and amplified from kidney homogenates of HA-I–positive mice. Primary kidney epithelial cells prepared from virus-free baby mice were used for amplification. Cytopathic effects typical of Py developed over a period of 7–12 d in every inoculated culture. First-round lysates gave plaque titers in the range of 10^6^–10^7^ pfu/ml. These titers were lower by 1–2 orders of magnitude compared with those of laboratory-adapted strains. Several isolates were tested and shown to be neutralized by anti-Vp1 antibody or by sera from laboratory mice infected by Py (unpublished data). The feral mouse virus isolates were further amplified and analyzed by DNA sequencing of selected regions of the viral genome. Six isolates from mice trapped at three locations were tested for their abilities to induce tumors in newborn mice (see further below).

### Vp1 Coding Sequences and Regulatory Regions of Py Isolates from Feral Mice

**Figure 2:**
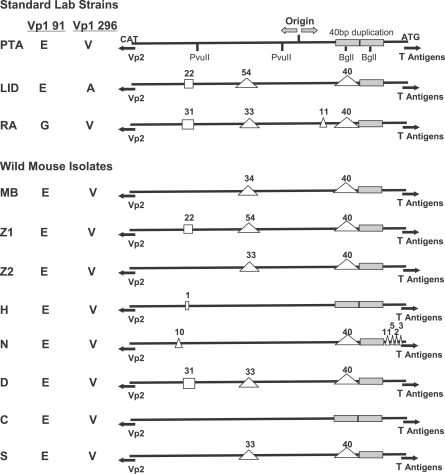
Origin Regions of Three Standard Polyoma Laboratory Strains and Eight Polyoma Isolates from Feral Mice Amino acids at positions 91 and 296 of Vp1 are shown on the left. The origin regions on the right are denoted schematically as horizontal lines representing the sequences between the start sites for Vp2 (CAT←) and the T antigens (ATG→). The PTA sequence on the top is used as a reference. The bidirectional replication origin (← →) and the PvuII and Bgl1 restriction sites are shown as points of reference. The 40–base pair duplication at the Bgl1 sites in PTA is designated with shaded rectangles. Insertions and deletions are denoted by open boxes and open triangles, respectively, along with the number of base pairs.

One virus isolate from each of the eight locations was chosen initially for sequence analysis (Figure 2). To determine the Vp1 types in these isolates, virus was concentrated from first or second passage lysates and analyzed by PCR and sequencing. A 1083-bp fragment covering 94% of Vp1 coding sequences was amplified and purified from each of the eight viruses. Internal primers were used to determine sequences flanking the 91 and 296 coding regions. All eight isolates encoded glutamic acid at position 91 and valine at 296. An additional seven viruses were analyzed from different mice taken from each of the sites where multiple animals were trapped. These seven isolates also encoded glutamic acid at 91 and valine at 296 in their Vp1. Thus, 15 of 15 isolates from mice trapped at eight different locations carried the polymorphisms found in standard large plaque tumorigenic laboratory strains represented by PTA as the prototype. The entire Vp1 coding regions of the original isolates were sequenced and confirmed to be identical to that of PTA.

Regulatory sequences flanking the replication origins of the prototype wild-type strains are variable compared with their highly conserved coding regions [28]. It was therefore of interest to determine the degree of variation in regulatory sequences of the new isolates. It should be noted that all three prototype strains were being propagated in our laboratory during the course of this investigation with the RA strain being most widely used. This raises the possibility of contamination in attempts to amplify and sequence the new virus isolates. To address this concern and to learn more about the new isolates, crude lysates were concentrated and used for analysis. The entire non-coding regions between the ATG of the T antigens on the early side of the replication origin and the ATG of Vp2 on the late side (ca. 450 bp) were amplified and sequenced in one isolate from each of the eight sites. Results are presented schematically in Figure 2 and the sequences are given in Figure S1.

Insertions, deletions, and single base changes distinguish the different isolates. The S and Z2 isolates were identical to each other but different from all three prototype strains. Differences in the new isolates were found dispersed throughout the non-coding regions in both the “A” and “B” enhancers and on the early side of the origin. PTA carries two copies of a 40-bp sequence around the Bgl1 site, while the related LID strain carries a single copy [9]. Two of the isolates (C and H) carried the duplication as found in PTA, and the other five (MB, Z1, Z2, D, and S) carried a single copy. Origin regions of several additional isolates derived from mice trapped at the same location were sequenced. Sequences for independent isolates from each of three sites examined were found to be the same as shown in Figure 2. Of the 15 isolates examined, only 1 (C) was found to be identical to PTA throughout the origin region. None matched either RA or LID. These results rule against contamination and support the conclusion that the isolates represent new strains.

### Tests for Tumor Induction by Py Isolates from Feral Mice

PTA, RA, and LID have been compared for their abilities to induce tumors following inoculation into newborn mice of the highly susceptible C3H/BiDa strain [8,9]. PTA induces multiple tumors in 100% of these animals. Tumors are of both epithelial and mesenchymal types. RA induces only mesenchymal tumors and at a much lower frequency and with longer average latency. The absence of epithelial tumors with RA is an important feature distinguishing RA from PTA [8]. LID at doses of approximately 10^5^ pfu/animal or higher kills C3H/BiDa within roughly two weeks. At much lower doses, animals survive and develop a tumor profile similar to that induced by PTA.

The finding of a uniform Vp1 type identical to PTA in the feral mouse isolates makes several predictions about the biological behavior of the new strains. First, they should induce tumors at high frequency and these should include epithelial as well as mesenchymal types. Second, because the isolates lack the virulence determinant of LID (296A), they should not cause early deaths (≤ 3 wk). To test these predictions, two isolates from each of three locations were inoculated into newborn C3H/BiDa mice. The results clearly support the predictions based on the importance of Vp1 type. All six isolates induced epithelial as well as mesenchymal tumors (Table 3). There were no early deaths. Latencies of tumor development, measured as the average age to necropsy, varied considerably but were longer than with PTA. The longer latencies are attributable to the lower titers of the new isolates. Latencies are known to increase as the titers decrease [8].

**Table 3:**
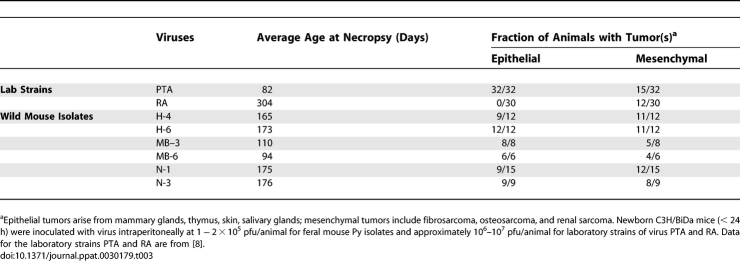
Tumor Induction by Laboratory Strains and Feral Mouse Isolates of Polyomavirus

## Discussion

Py establishes a silent persistent infection in natural populations of mice, yet behaves in the laboratory as a powerful oncogenic agent (reviewed in [4]). This discordance could be explained if naturally occurring virus strains were attenuated in some manner compared with highly oncogenic strains used in the laboratory. Variations in the major capsid protein of Py dictate a wide range of biological properties upon inoculation into newborn mice, ranging from non-pathogenic to tumorigenic to virulent. The purpose of the present investigation was to determine if these variations, presently known only in laboratory strains of virus, are also found in nature. Fifteen new isolates of virus were derived from feral mice trapped at eight different locations. Only a single type of Vp1 was found, and it matches that of the large plaque highly oncogenic laboratory strain PTA [8,14]. The finding of PTA-like Vp1 sequences in new isolates is not due to rapid selection during passage in culture, as both LID and RA are routinely and faithfully passaged under the same conditions without change in their Vp1s. Six of six of the new isolates tested proved to be highly tumorigenic with a tissue tropism similar to that of PTA. Natural selection therefore results in virus that retains its pathogenic potential. Natural variants of the virus have adopted a “middle ground” with respect to their common Vp1 type and ability to spread in the natural host. Their oncogenic potential is effectively offset by protective maternal antibody and possibly other host or environmental factors that accompany natural transmission (reviewed in [4]).

Glutamic acid at position 91 and valine at position 296 define critical features of this common Vp1. Glutamic acid–91 is critical for discriminating between true receptors carrying a terminal unbranched sialic acid and pseudoreceptors with branched chain sialic acids [5,11,12]. A glycine at this position allows binding to pseudoreceptors, leading to significant attenuation of virus spread [10]. No naturally occurring strains of Py with glycine-91 have been found. The failure to find such strains implies that binding to pseudoreceptors leads to an inhibition of virus spread to a degree that is incompatible with natural transmission. Therefore, binding to branched chain sialic acids should not be viewed as a natural means for attenuating the pathogenic potential of the virus.

Valine at position 296 establishes a hydrophobic contact with the receptor, decreasing the rate of dissociation and spread of the virus. Substitution of alanine at this position results in loss of the hydrophobic contact and a decreased overall avidity of binding [9]. The magnitude of the change in avidity has not been determined precisely. However, given the engagement of multiple capsomeres (each a pentamer of Vp1) at the cell surface, even a modest decrease in affinity would be expected to confer a significantly lower overall avidity of virus binding to the cell. The alanine substitution clearly results in more rapid and extensive virus spread and results in a lethal infection of newborn mice [9,10]. No variants with this substitution have been found in nature. This suggests the importance of control of the rate of virus dissociation from infected cell debri and subsequent spread within the newly infected host. Given the importance of glutamic acid at 91 in allowing efficient spread without interference from pseudoreceptors, the coupling with valine as opposed to alanine at 296 would appear to be essential because the latter would confer a degree of virulence potentially incompatible with host survival.

Some viruses promote their release and spread through mechanisms that effectively destroy host cell receptors. Prominent examples are the neuraminidase of influenza viruses and the *Vpu* and *nef* proteins of HIV and simian immunodeficiency virus which act directly or indirectly to destroy cell receptors. For Py and perhaps other viruses that lack the means to destroy their own receptors, it appears to be critical that they regulate their receptor-binding properties within narrow limits. Affinity of binding must be high enough to promote efficient cell attachment and entry, yet not so high as to inhibit dissociation from cell debris and prevent virus spread. At the same time, receptor affinity must not be so low as to allow rapid dissociation and spread, which could endanger the life of the host. The properties of Py strains RA and LID, lying outside these limits, serve to illustrate the constraints of natural selection.

While Vp1-coding sequences were entirely conserved, regulatory sequences showed considerable variability among the new virus isolates. This variability was evident comparing isolates from different locations. In a limited sampling of multiple virus isolates from the same location, regulatory sequences were conserved, indicating relative stability of virus circulating at a given time within the same host breeding population. This finding is consistent with the epidemiology and natural history of infection in wild mice ([16,26,27] and reviewed in [4]).

The origin and biological significance of the variability in regulatory sequences of the new isolates are not clear. Sequences at the replication origin per se as well as the binding sites for the large T antigen are conserved among the feral mouse isolates and laboratory strains of virus, although the number of large T binding sites varies. Signaling pathways from the middle and small T antigens converge on the enhancer regions at different sites via a number of cellular factors affecting viral DNA replication as well as transcription [29,30]. Different enhancer sequences play roles in the host range properties of the virus in cell culture [30–35] and in the animal [36–38]. Particular features of the tumor profile are controlled by a 40-bp duplication upstream of the early promoter [39]. Studies of the related pneumotropic polyoma virus of mice have provided evidence for incorporation of host sequences into the viral enhancers [40,41]. Further studies of the new Py isolates and their molecularly cloned derivatives will be required to determine whether and to what extent different enhancer sequences may dictate differences in tissue tropism for replication and induction of tumors.

## Materials and Methods

### Trapping and serological testing.

Feral mice (Mus musculus) from 12 locations in and around Boston and as far away as Cuttyhunk Island, MA, and New York City were trapped and recovered alive using Sherman Traps and mouse chow coated with peanut butter as bait. Mice were humanely killed, sera collected for serological testing, and kidneys frozen at −80 °C. One kidney from each animal was sent to the Laboratory of Comparative Medicine at Yale University School of Medicine for testing of common mouse pathogens before attempting virus isolation in our laboratory. Results on all mice were negative for all agents tested, including: hantavirus; mouse parvovirus; lymphocytic choriomeningitis virus; Theiler's mouse encephalomyelitis virus; Sendai virus; minute virus of mice; mouse hepatitis virus; ectromeila; reovirus; and mycoplasma.

Serological testing for Py was performed in our laboratory using a standard HA-I assay in V-shaped 96-well microtiter plates. Serial 2-fold dilutions of mouse sera were incubated with an equal volume of 2–4 HA units of wild-type polyoma (PTA strain) for 30 min at 37 °C, followed by addition of a suspension of guinea pig erythrocytes (2 × 10^7^/ml). Further incubation was at 4 °C for a minimum of 6 h. HA-I titer was scored as the reciprocal of the highest dilution of serum that prevented agglutination.

### Virus isolation.

Mice with sera showing HA-I titers of ≥ 160 were judged to be positive and were considered likely carriers of the virus. Frozen kidneys from these HA-I–positive mice were thawed and homogenized in 2 ml serum-free Dulbecco's Modified Eagle's Medium. Aliquots of the homogenates were used to inoculate primary baby mouse kidney epithelial cells prepared from Py-free baby ICR mice. Virus lysates from these cultures were titered by plaque assay on NIH3T3 cells, concentrated by pelleting, and resuspended for further analysis.

### PCR and DNA sequence analysis.

Viral DNA was extracted from crude or concentrated virus. Selected regions of the viral genome were amplified by PCR and sequenced. For Vp1, a 1083-bp region covering most of the Vp1 coding sequence was first amplified [5′ GAATATAGCTGAATACACAG3′ (sense) and 5′ AGGTGCTGGACCTTGTGACAGGG3′ (anti-sense)] and then sequenced using primers 5′ AACAGTGAGCCAGAGCCCACCACC3′ for the 91 region and 5′ GAGGGAGGCCATGGGATAGGG3′ for the 296 region. Additional sequencing was carried out to confirm and extend coverage of the entire Vp1. The origin regions were amplified with primers 5′ GTACCGCTGTATTCCTAG3′ and 5′ CATTCTCAGATTGTATACTTCAG3′ giving a fragment of 1061 bp encompassing all of the non-coding sequences between the ATG of Vp2 on the late side and the ATG for the T antigens on the early side of the origin. Sequencing of this amplicon was carried out with primers 5′ CTCATTACACCCTCCAAAGTC3′ and 5′ CATTCTCAGATTGTATACTTCAG3′. Amplicons were sequenced using cycle sequencing with BigDye v3.1 dideoxy fluorescent terminators and a 3730xL DNA Analyzer from Applied Biosystems.

### Tumor profile studies.

Tumor induction studies were carried out with six of the 15 virus isolates following previously described procedures [8]. Briefly, newborn C3H/BiDa mice (< 24 h old) were inoculated with virus intraperitoneally at approximately 10^5^ pfu/animal. Six to 12 animals were used for each virus. Animals were followed for tumor development grossly and were necropsied when moribund. Tumors were confirmed by histological examination and grouped as epithelial or mesenchymal. The former includes tumors arising from mammary glands, thymus, skin, and salivary glands and the latter includes fibrosarcoma, osteosarcoma, and renal sarcoma. Results with the six new isolates are compared with previously published results for PTA and RA [8].

## Supporting Information

Figure S1Nucleotide Sequences of the Noncoding Regions of Prototype Laboratory Strains (PTA, RA, and LID) and New Isolates of Polyoma Viruses (MB, Z1, Z2, H, N, D, C, and S)The sequences cover the region from the translation start site for Vp2 on the late side (nucleotide 5002; NCBI accession number J02288 for polyomavirus strain A2) to the translation start site of the T antigens on the early side (nucleotide 177 in A2). The ori-core sequence (nucleotides 5276–44); [42] is boxed. 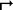 marks nucleotide 1 in A2. Two PvuII sites (nucleotides 5130–5135 and 5267–5272 in A2) and one BglI site (nucleotides 89–99 in A2) are shaded for reference. All sequences are compared with PTA, which has a 40–base pair duplication on the early side of the replication origin (nucleotides 71–110 in A2, not duplicated). Variations from the PTA sequence are shown in red.
(28 KB PDF)

### Accession Numbers

GenBank (http://www.ncbi.nlm.nih.gov/Genbank/index.html) accession numbers for sequences of laboratory strains of polyoma virus are as follows: A2 strain (JO2288), PTA strain (U27812), LID strain (U27813), and RA strain Vp1 (M34958). Additional sequences for non-coding and coding regions of RA may be found in [14] and [28].

## Abbreviations

HA-I: hemagglutination inhibition
Py: murine polyoma virus
T: tumor
