# Supplementary material for: Receptor-Binding and Oncogenic Properties of Polyoma Viruses Isolated from Feral Mice
Source: PLoS Pathog. 2007 Dec 14;3(12):e179. doi: 10.1371/journal.ppat.0030179 (PMC2134959; doi:10.1371/journal.ppat.0030179)
Supplement: Supplementary file 1 [file ppat.0030179.sg001.pdf]

Supplementary Figure 1. Nucleotide sequences of the noncoding regions of prototype laboratory strains (PTA, RA and LID) and new isolates of polyoma viruses (MB, Z1, Z2, H, N, D, C, and S).

```
PTA    CATTTTGAAA ATTCACTTAC TTGATCAGCT TCAGAAGATG GCGGAGGGCC TCCAACACAG TAATTTTCCT
LID    CATTTTGAAA ATTCACTTAC TTGATCAGCT TCAGAAGATG GCGGAGGGCC TCCAACACAG TAATTTTCCT
RA     CATTTTGAAA ATTCACTTAC TTGATCAGCT TCAGAAGATG GCGGAGGGCC TCCAACACAG TAATTTTCCT
MB     CATTTTGAAA ATTCACTTAC TTGATCAGCT TCAAAAGATG GCGGAGGGCC TCCAACACAG TAATTTTCCT
Z1     CATTTTGAAA ATTCACTTAC TTGATCAGCT TCAGAAGATG GCGGAGGGCC TCCAACACAG TAATTTTCCT
Z2     CATTTTGAAA ATTCACTTAC TTGATCAGCT TCAGAAGATG GCGGAGGGCC TCCAACACAG TAATTTTCCT
H      CATTTTGAAA ATTCACTTAC TTGATCAGCT TCAGAAGATG GCGGAGGGCC TCCAACACAG TAATTTTCCT
N      CATTTTGAAA ATTCACTTAC TTGATCAGCT TCAGAAGATG GCGGAGGGCC TCCAACACAG TAATTTTCCT
D      CATTTTGAAA ATTCACTTAC TTGATCAGCT TCAGAAGATG GCGGAGGGCC TCCAACACAG TAATTTTCCT
C      CATTTTGAAA ATTCACTTAC TTGATCAGCT TCAGAAGATG GCGGAGGGCC TCCAACACAG TAATTTTCCT
S      CATTTTGAAA ATTCACTTAC TTGATCAGCT TCAGAAGATG GCGGAGGGCC TCCAACACAG TAATTTTCCT

PTA    CCCGACTCTT AAAATAGAAA ATGTCAAGTC AGTTAAGCAG GAAG------ ---------- ----------
LID    CCCGACTCTT AAAATAGAAA ATGTCAAGTC AGTTAAGCAG GAAGTCACTA GCTGACCGCA GCTGA-----
RA     CCCGACTCTT AAAATAGAAA ATGTCAAGTC AGTTAAGCAG GAAGTGACTA ACTGACCGCA GCTGGCCGTG
MB     CCCGACTCTT AAAATAGAAA ATGTCAAGTC AGTTAAGCAG GAAG------ ---------- ----------
Z1     CCCGACTCTT AAAATAGAAA ATGTCAAGTC AGTTAAGCAG GAAGTCACTA GCTGACCGCA GCTGAG----
Z2     CCCGACTCTT AAAATAGAAA ATGTCAAGTC AGTTAAGCAG GAAG------ ---------- ----------
H      CCCGACTCTT AAAATAGAAA ATGTCAAGTC AGTTAAGCAG GAA-TGC--- ---------- ----------
N      CCCGACTCTT AAAATAGAAA ATGTC----- -----AGCAG GAA-TG---- ---------- ----------
D      CCCGACTCTT AAAATAGAAA ATGTCAAGTC AGTTAAGCAG GAAGTCACTA GCTGACCGCA GCTGGCCGTG
C      CCCGACTCTT AAAATAGAAA ATGTCAAGTC AGTTAAGCAG GAAG------ ---------- ----------
S      CCCGACTCTT AAAATAGAAA ATGTCAAGTC AGTTAAGCAG GAAG------ ---------- ----------

                                PvuII
PTA    -----TCACT AGCTGACCGC AGCTGGCCGT GCGACATCCT CTTTTAATTA GTTGCTAGGC AACTGGCCTC
LID    ----GTCACT AGCTGACCGC AGCTGGCCGT GCGACATCCT CTTTTAATTA GTTGCTAGGC AACTGCCCTC
RA     CGACATGACT AACTGACCGC AGCTGGCCGT GCGACATCCT CTTTTAATTA GTTGCTAGGC AACTGCCCTC
MB     -----TCACT AGCTGACCGC AGCTGGCCGT GCGACATCCT CTTTTAATTA GTTGCTAGGC AACTGGCCTC
Z1     -----TCACT AGCTGACCGC AGCTGGCCGT GCGACATCCT CTTTTAATTA GTTGCTAGGC AACTGCCCTC
Z2     -----TGACT AACTGACCGC AGCTGGCCGT GCGACATTCT CTTTTAATTA GTTGCTAGGC AACTGCCCTC
H      ------CGCT AACTGACCGC AGCTGGAAGT GCGTCATCCT CTTTTAATTA GTTGCTAGGC AACTGGCCTC
N      ------CACT AGCTGACCGC AGCTGGCCGT GCGTCATCCT CTTTTAATTA GTTGCTAGGC AACTGGCCTC
D      CGACATGACT AACTGACCGC AGCTGGCCGT GCGACATCCT CTTTTAATTA GTTGCTAGGC AACTGCCCTC
C      -----TCACT AGCTGACCGC AGCTGGCCGT GCGACATCCT CTTTTAATTA GTTGCTAGGC AACTGGCCTC
S      -----TGACT AACTGACCGC AGCTGGCCGT GCGACATTCT CTTTTAATTA GTTGCTAGGC AACTGCCCTC

PTA    CAGAGGGCAG TGTGGTTTTG CAAGAGGAAG CAAAAAGCGG CAGTGTGGTT TTGCAAGAGG AAGCAAAAAG
LID    CAGAGGGCAG TGTGGTTTTG CAAGAGGAAG CAA------- ---------- ---------- ----------
RA     CAGAGGGCAG TGTGGTTTTG CAAGAGGAAG CAAAAAG--- ---------- ---------- ----------
MB     CAGAGGGCAG TGTGGTTTTG CAAGAGGAAG CAAAAAG--- ---------- ---------- ----------
Z1     CAGAGGGCAG TGTGGTTTTG CAAGAGGAAG CAA------- ---------- ---------- ----------
Z2     CAGAGGGCAG TGTGGTTTTG CAAGAGGAAG CAAAAAG--- ---------- ---------- ----------
H      CAGAGGGCAG TGTGGTTTTG CAAGAGGAAG CAAAAAGCGG CAGTGTGGTT TTGCAAGAGG AAGCAAAAAG
N      CAGAGGGCAA TGTGGTTTTG CAAGAGGAAG CAAAAAGCGG CAGTGTGGTT TTGCAAGAGG AAGCAAAAAG
D      CAGAGGGCAG TGTGGTTTTG CAAGAGGAAG CAAAAAG--- ---------- ---------- ----------
C      CAGAGGGCAG TGTGGTTTTG CAAGAGGAAG CAAAAAGCGG CAGTGTGGTT TTGCAAGAGG AAGCAAAAAG
S      CAGAGGGCAG TGTGGTTTTG CAAGAGGAAG CAAAAAG--- ---------- ---------- ----------

                                                           PvuII
PTA    CCTCTCCACC CAGGCCTAGA ATGTTTCCAC CCAATCATTA CTATGACAAC AGCTGTTTTT TTTAGTATTA
LID    ---------- -------AGA ATGTTTCCAC CCACTCATTA CTATGACAAC AGCTGTTTTT TTTAGTATTA
RA     CCTCTCCACC CAGGCCTAGA ATGTTTCCAC CCAATCATTA CTATGACAAC AGCTGTTTTT TTTAGTATTA
MB     -CTCTCCACC CAGGCCTAGA ATGTTTCCAC CCAATCATTA CTATGACAAC AGCTGTTTTT TTTAGTATTA
Z1     ---------- -------AGA ATGTTTCCAC CCAATCATTA CTATGACAAC AGCTGTTTTT TTTAGTATTA
```

```
Z2     CCTCTCCACC CAGGCCTAGA ATGTTTCCAC CCAATCATTA CTATGACAAC AGCTGTTTTT TTTAGTATTA
H      CCTCTCCACC CAGGCCTAGA ATGTTTCCAC CCAATCATTA CTATGACAAC AGCTGTTTTT TTTAGTATTA
N      CCTCTCCACC CAGGCCTAGA ATGTTTCCAC CCAATCATTA CTATGACAAC AGCTGTTTTT TTTAGTATTA
D      CCTCTCCACC CAGGCCTAGA ATGTTTCCAC CCAATCATTA CTATGACAAC AGCTGTTTTT TTTAGTATTA
C      CCTCTCCACC CAGGCCTAGA ATGTTTCCAC CCAATCATTA CTATGACAAC AGCTGTTTTT TTTAGTATTA
S      CCTCTCCACC CAGGCCTAGA ATGTTTCCAC CCAATCATTA CTATGACAAC AGCTGTTTTT TTTAGTATTA

                         Ori-core
PTA    AGCAGAGGCC GGGGGCCCCT GGCCTCCGCT TACTCTGGAG AAAAAGGAGA GAGGCATTGT AGAGGCTTCC
LID    AGCAGAGGCC GGGGGCCCCT GGCCTCCGCT TACTCTGGAG AAAAAGAAGA GAGGCATTGT AGAGGCTTCC
RA     AGCAGAGGCC GGGGGCTCCT GGCCTCCGCT TACTCTGGAG AAAAAGAAG- ---------- AGAGGCTTCC
MB     AGCAGAGGCC GGGGGCCCCT GGCCTCCGCT TACTCTGGAG AAAAAGGAGA GAGGCATTGC AGAGGCTTCC
Z1     AGCAGAGGCC GGGGGCCCCT GGCCTCCGCT TACTCTGGAG AAAAAGAAGA GAGGCATTGC AGAGGCTTCC
Z2     AGCAGAGGCC GGGGGCCCCT GGCCTCCGCT TACTCTGGAG AAAAAGAAGA GAGGCATTGC AGAGGCTTCC
H      AGCAGAGGCC GGGGGCCCCT GGCCTCCGCT TACTCTGGAG AAAAAGGAGA GAGGCATTGT AGAGGCTTCC
N      AGCAGAGGCC GGGGGCCCCT GGCCTCCGCT TACTCTGGAG AAAAAGAAGA GAGGCATTGT AGAGGCTTCC
D      AGCAGAGGCC GGGGGCCCCT GGCCTCCGCT TACTCTGGAG AAAAAGGAGA GAGGCATTGT AGAGGCTTCC
C      AGCAGAGGCC GGGGGCCCCT GGCCTCCGCT TACTCTGGAG AAAAAGGAGA GAGGCATTGT AGAGGCTTCC
S      AGCAGAGGCC GGGGGCCCCT GGCCTCCGCT TACTCTGGAG AAAAAGAAGA GAGGCATTGT AGAGGCTTCC

                                       BglI
PTA    AGAGGCAACT TGTCAAAACA GGACTGGCGC CTTGGAGGCG CTGTGGGGCT TGTCAAAACA GGACTGGCGC
LID    AGAGGCAACT ---------- ---------- ---------- ---------- TGTCAAAACA GGACTGGCGC
RA     AGAGGCAACT ---------- ---------- ---------- ---------- TGTCAAAACA GGACTGGCGC
MB     AGAGGCAACT ---------- ---------- ---------- ---------- TGTCAAAACA GGACTGGCGC
Z1     AGAGGCAACT ---------- ---------- ---------- ---------- TGTCAAAACA GGACTGGCGC
Z2     AGAGGCAACT ---------- ---------- ---------- ---------- TGTCAAAACA GGACTGGCGC
H      AGAGGCAACT TGTCAAAACA GGACTGGCGC CTTGGAGGCG CTGTGGGGCT TGTCAAAACA GGACTGGCGC
N      AGAGGCAACT ---------- ---------- ---------- ---------- TGTCAAAACA GGACTGGCGC
D      AGAGGCAACT ---------- ---------- ---------- ---------- TGTCAAAACA GGACTGGCGC
C      AGAGGCAACT TGTCAAAACA GGACTGGCGC CTTGGAGGCG CTGTGGGGCT TGTCAAAACA GGACTGGCGC
S      AGAGGCAACT ---------- ---------- ---------- ---------- TGTCAAAACA GGACTGGCGC

         BglI
PTA    CTTGGAGGCG CTGTGGGGCC ACCCAAATTG ATATAATTAA GCCCCAACCG CCTCTTCCCG CCTCATTTCA
LID    CTTGGAGGCG CTGTGGGGCC ACCCAAATTG ATATAATTAA GCCCCAACCG CCTCTTCCCG CCTCATTTCA
RA     CTTGGAGGCG CTGTGGGGCC ACCCAAATTG ATATAATTAA GCCCCAACCG CCTCTTCCCG CCTCATTTCA
MB     CTTGGAGGCG CTGTGGGGCC ACCCAAATTG ATATAATTAA GCCCCAACCG CCTCTTCCCG CCTCATTTCA
Z1     CTTGGAGGCG CTGTGGGGCC ACCCAAATTG ATATAATTAA GCCCCAACCG CCTCTTCCCG CCTCATTTCA
Z2     CTTGGAGGCG CTGTGGGGCC ACCCAAATTG ATATAATTAA GCCCCAACCG CCTCTTCCCG CCTCATTTCA
H      CTTGGAGGCG CTGTGGGGCC ACCCAAATTG ATATAATTAA GCCCCAACCG CCTCTTCCCG CCTCATTTCA
N      CTTGGAGGCG CTGTGGGGCC ---------- -TATAATAAA -----AA--G AC---TGCCG CCTCATTTCA
D      CTTGGAGGCG CTGTGGGGCC ACCCAAATTG ATATAATTAA GCCCCAACCG CCTCTTCCCG CCTCATTTCA
C      CTTGGAGGCG CTGTGGGGCC ACCCAAATTG ATATAATTAA GCCCCAACCG CCTCTTCCCG CCTCATTTCA
S      CTTGGAGGCG CTGTGGGGCC ACCCAAATTG ATATAATTAA GCCCCAACCG CCTCTTCCCG CCTCATTTCA

PTA    GCCTCACCAC CATCATG
LID    GCCTCACCAC CATCATG
RA     GCCTCACCAC CATCATG
MB     GCCTCACCAC CATCATG
Z1     GCCTCACCAC CATCATG
Z2     GCCTCACCAC CATCATG
H      GCCTCACCAC CATCATG
N      GCCTCACCAC CATCATG
D      GCCTCACCAC CATCATG
C      GCCTCACCAC CATCATG
S      GCCTCACCAC CATCATG
```
